# Extend the ProFamy cohort-component method to conduct probabilistic households and living arrangement projections

**DOI:** 10.1007/s42379-024-00171-6

**Published:** 2024-12-30

**Authors:** Yi Zeng, Zhenglian Wang, Qiushi Feng, Danan Gu, Junni Zhang, Wei Tang, Kenneth Land

**Affiliations:** 1 National School of Development, Peking University, Beijing, China; 2 Center for Study of Aging and Human Development and Geriatrics Division, School of Medicine, Duke University, Durham, NC USA; 3 China Population and Development Research Center, Beijing, China; 4 Department of Sociology and Centre for Family and Population Research, National University of Singapore, Singapore, Singapore; 5 United Nations Population Division, New York, USA; 6 Duke University Population Research Institute, Durham, USA

**Keywords:** Household and living arrangement projections, ProFamy methods, Deterministic and probabilistic projections, Probabilistic household and living arrangement projections (PHPs)

## Abstract

In this commentary, we first briefly review the significant utilities of household and living arrangement projections and the main types of methods for conducting household projections. In the second and third sections, we summarize basic ideas, data needed, assessments and applications of ProFamy extended cohort-component methods/software for households and living arrangement projections; and we emphasize the importance to extend the ProFamy methods and software from deterministic to probabilistic households and living arrangement projections. In section 4, we demonstrate that the ProFamy approach provides an adequate and highly feasible modelling framework to extend probabilistic households and living arrangement projections (PHPs), in which the population size/structure projection outcomes are in consistence with those of probabilistic population projections (PPPs) released by United Nations Population Division (UNPD). In the last Section, we discuss and recommend applying the user-friendly R package DemoRates of ProFamy software to estimate rural/urban (or race)-sex-age-specific standard schedules and the demographic summary measures, to conduct analyses and projections, such as single-parent households, caregivers, and care needs/costs for disabled older adults, age-friendly housing and households-based energy demands, etc. for healthy aging and sustainable development studies. Finally, we discuss the prospects of our ongoing international collaborative research project to substantially extend ProFamy cohort-component method from deterministic into probabilistic households and living arrangement projection (PHPs). As compared with ProFamy deterministic projection method, the PHPs produces a lot of additional outcomes of probabilistically projected households and living arrangements in 2021–2100 with uncertainty intervals that are crucial for healthy aging and sustainable development studies.

## Why projections of households and living arrangements are important

Household and living arrangement projections are useful in socioeconomic and welfare planning. For example, several welfare programs in the Europe and the United States restrict eligibility to single-parent (especially single-mother) households (Maldonado & Nieuwenhuis, 2015). As a result, projecting the needs and costs of such programs depends heavily upon projections of the numbers, types, and sizes of single-parent households and children living in these households in the future (Casey & Maldonado, 2012). In addition to disability status, households and living arrangements are the major determinants of the amounts, types and use of long-term care for the older adults (Francesca et al., 2011). Clearly, projections of households and living arrangements that include older adults’ marital/union statuses, number of surviving children and co-residence with children are significantly useful to face the challenges of rapid population aging in all countries in the world (Zeng et al., 2014).

Compared with changes in population sizes, changes in numbers, sizes and structures of households are more important in analyses on energy-related consumption, human impacts on the environment, and sustainable development (Bradbury et al., 2014). This is because energy-related commodities such as water, electricity, gas and vehicles are often purchased and consumed by households rather than individuals. Chen et al. (2022) discovered that residential energy use intensity is significantly influenced by housing characteristics and socio-demographic factors. In the United States, millions of households consume huge amounts of home-based energy use and disaggregated thermostatically (Thorve et al., 2023). In the Republic of Korea, the household sector was responsible for about 52% of the national primary energy consumption in 1980–2000 (Park & Heo, 2007). Air pollution, both indoors and outdoors, is closely related to household energy consumption, especially in developing countries where clean fuels are not widely available (IEA, 2004); and households are responsible for 72% of global greenhouse gas emissions (Dubois et al., 2019).

Changing demographic factors (including lower fertility and fewer children, separation or divorce of married couples, increasing incomes leading to increases in housing, purchase/rental of second homes or decreases in housing by households; more migrations, and vanishing social norms that prescribe co-residence of old parents and adult children) contribute to smaller household size, and continuously and quickly increasing numbers of households all over the world (Gu et al., 2015). Pawlak et al. (2021) warned that elderly people have a higher demand for heating and electricity due to a less active lifestyle. In the past, aggregated energy use models based on household archetypes were typically used to relate to energy footprints, but in today’s world, there is urgent needs for more dynamic residential energy demand forecasts. Bardazzi and Pazienza (2017) pointed out that an aging population may hinder public policy efforts to reduce energy intensity and greenhouse gas emissions.

Clearly, it would seriously underestimate future years’ energy demands and mislead the policy makers, if the energy-use forecasting is based on population growth, which has slowed down greatly and already or will become negative soon in many countries. For example, the projections based on population changes seriously under-estimates the future residential energy demands in the Hebei province of China (Zeng et al., 2021; Fig. 1). Population growth worldwide is already greatly slowed down, and a new problem has arisen associated with a decline in fertility to the “lowest-low” level—a tendency formed to the negative natural population change in many countries (Korotayev et al., 2023). However, the number of households which truly determines home-based energy demands will continue to substantially increase in the next four decades. Clearly, conducting household projections by types and sizes is crucially important in forecasting energy demands and strategic planning of environmental protections, and considering population changes only would seriously under-estimate future energy demands and mislead policy makers (Bradbury et al., 2014; Keilman, 2003; Zeng et al., 2021).

Business market analyses of various home-based goods and services heavily rely on projections of households and living arrangements. An obvious example is that planning for housing needs and real estate market studies depend on projected numbers, types, and sizes of households in future years (Smith et al., 2008, 2012). The demand for households financing (Zhu, 2021), consumer durables such as appliances, furniture and vehicles are also determined by projected changes in household size and structure (e.g. Feng et al., 2020; Prskawetz et al., 2004).

Since the late 1990s, an increasing number of researchers and policymakers have demanded household projections at sub-national levels such as provinces (or states), counties and cities, and other small areas. Household and living arrangement projections at sub-national and county/city levels are useful for distributing government funds, allocating various types of resources, planning the development of infrastructure and public facilities, market research, production planning for household-related goods and services, and decisions on the expansion or reduction of local businesses (Swanson & Pol, 2009).

## The three main methods for projecting households

Demographers conduct household projections based primarily on three major types of methods: headship-rate, microsimulation, and macrosimulation (Willekens, 2010; Zeng et al., 2014; Page 10–12), which will be briefly reviewed and summarized here. Microsimulation models simulate life course events and keep detailed records of demographic status transitions for each individual of the sample population (Hammel, 2005; Wachter, 1987). Macrosimulation models deal with individuals grouped by specified attributes (e.g. with the same race, sex, age, and marital status) and the calculations proceed iteratively, group-by-group and time-period-by-time-period.

Comparisons between microsimulation and macrosimulation approaches for household projections can be found in the literature (e.g. Keilman, 2018; Van Imhoff & Keilman, 1992). The choice between a micro or macro model depends on the complexity of the researchers’ task and data availability. For detailed analyses of behavioural patterns and complex family kinship relationships, a microsimulation approach may be preferable, if the requested very detailed datasets are available. For relatively straightforward demographic analyses and household projections based on commonly available data for the purposes of policy analyses, market trends studies, and socioeconomic planning, especially projections used by non-experts, a macrosimulation approach may well be satisfactory.

The macrosimulation models, which rely on transition probabilities among various statuses of households, require data that have to be collected in special surveys or population registers. However, such data sources are not available in most countries, including the United States, Canada, and most European countries. In such household-status-transition models, there is no projection information for groups other than those based on the pre-defined state space. For instance, it is not possible to construct the distribution of households by size unless the size of the households is explicitly incorporated in the state space (Van Imhoff et al., 1995: 348). Incorporating both household size and types in the state space would largely increase the number of household statuses in the transition probability matrix that requires estimates at each age for males and females, which is likely not feasible in practical applications. Furthermore, the household type/status-transition-based model cannot directly link changes in household structure to demographic rates. For example, the probability of transition from the household status of married living with spouse and one or more children to not-married without children jointly depends on changes in divorce rates, death rates of spouse, fertility rates, and rates of children’s leaving the parental home. How these demographic rates are linked to the household status transition probabilities cannot be clarified using a household type/status-transition-based model.

The traditional headship-rate is computed by dividing the number of persons who are heads (or householders) of households by the total number of persons of the same age and sex. The numbers of households in future years are projected by extrapolating the headship-rates. The headship-rate method has been criticized widely by demographers for more than three decades (Grundy, 2013; Wilson, 2013; Mason & Racelis, 1992; Murphy, 1991; Spicer et al., 1992). Many demographers’ criticisms of the headship-rate method can be summarized into three main points. First, the headship-rate method is based on the vague and ill-defined concept of household heads, which leads to the phenomenon that if the census/survey interviews are conducted in the evenings or daytimes, the husbands or wives who answer the questions may more likely report themselves as household heads (Mason & Racelis, 1992; Murphy, 1991). Second, no direct link exists between headship rates and demographic rates. This makes it impossible to incorporate projected or assumed changes in marriage, divorce, fertility, children leaving parental home, migration, and deaths, which are the main drivers of household changes (Mason & Racelis, 1992; Spicer et al., 1992). Third, the information on households produced by headship-rate projections is very limited, it lumps all household members other than heads into one category “non-head” with no projected information, and thus inadequate for detailed planning and analysis (Bell & Cooper, 1990; Wilson, 2013). This limitation makes it impossible to study the household status and living arrangements of the older adults, young and middle-age adults and children, who are not heads of households. Consequently, the headship-rate method is not appropriate for projecting home-based care needs and costs of disabled older adults, which are directly linked to all older adults who may or may not be the heads of the households. The propensity method, which is the extension of the headship-rate, avoids the first drawback but it still suffers the second and third drawbacks of the headship-rate method (Wilson, 2013).

## The profamy extended cohort-component method and applications

### A brief introduction to the ProFamy method

Benefiting from methodological advances in multistate demography (Land & Rogers, 1982; Willekens et al., 1982), Bongaarts (1987) innovatively developed a nuclear-family-status life table model. Zeng (1986, 1991) extended Bongaarts’ nuclear-family-status life table model into a general- family-status life table model that includes both nuclear and three-generation family households. Based on Bongaarts’s and Zeng’s one-sex life table models, Zeng et al. (1998) initially proposed and Zeng et al. (2006, 2013) further developed the ProFamy model, which is a two-sex multistate cohort-component method that projects numbers of individuals by sex, age and statuses of marriage/union, numbers of co-residing children and parents, and derives household types/sizes from these projected individuals’ statuses. The ProFamy extended cohort-component methods/software projects simultaneously the households of various types and sizes (Ref. Table 2 in Zeng et al., 2006), and sex, age, marital/union status, numbers of co-residing parents and children distributions of all individual members of the population under study. The projection outcome of the population age/sex distributions produced by ProFamy approach are fully consistent with those produced using the cohort-component population projection approach, as long as both approaches use the same input data of fertility, mortality and migration, while the ProFamy approach produces additional information on households and living arrangements in the future years. Clearly, the ProFamy method overcomes the serious shortages of the traditional headship-rate method, that is not linked to demographic rates, projects only a few household types without size information, and deals with household “heads” but not other household members.

The ProFamy model applies the harmonic mean (Keilman, 1985) to ensure consistencies between the two sexes and between children and parents. More specifically, the numbers of newly married and divorced males each year are adjusted to equal the numbers of newly married and divorced females, and numbers of children who leave (or return to) the parental homes each year are adjusted to equal to the corresponding numbers of parents who experience the changes in the status of number of co-residing children (Zeng et al., 1998).

### Input data needed to use ProFamy method for household and living arrangement projections

Table 1 presents the data needed to project households and living arrangements using the ProFamy extended cohort-component method, with comparisons to population projections, including the probabilistic population projections (PPPs) for each of all countries worldwide by the United Nations Population Division (UNPD). As commented by Willekens (2010: 11), a major strength of the ProFamy model is that family and household dynamics are derived and projected from demographic events, and consequently, it requires conventional demographic data that are available from ordinary surveys, censuses, vital statistics or population registers (see Table 1). Because its reliance on commonly available demographic rates, ProFamy provides a useful tool to quantitatively assess the effects of demographic changes in marriages, divorces, fertility, mortality, migrations, etc., on households and living arrangement dynamics (Willekens, 2010). In fact, as demonstrated in Table 1, the majority of the needed data for household and living arrangement projections using the ProFamy methods/software are the same as the needed data for population projections (including the PPPs). The main additional work of data preparation for household and living arrangement projections using the ProFamy methods/software is minimal. It namely needs to estimate age-parity-specific occurrence/exposure (o/e) rates of marital and non-marital fertility and age-sex-specific o/e rates and total rates of marriages and divorces, which will be discussed in more details later in Sect. 3.6.

**Table 1:** Data needed to project households and living arrangements using the ProFamy extended cohort-component method, with a comparison to population projections, including United Nations Population Division (UNPD) Probabilistic Population Projections (PPPs)

Contents of the needed data for the projections	ProFamy household projection	Population projections (incl. PPPs)
**(1) Baseline population of starting year of projection at national or sub-national level**		
(a) A census micro sample or population register or an exceptionally large survey data file with only a few needed demographic variables, including sex, age, marital/union status, relationship to the householder, and living in a private or institutional household;	√ (and a few more available variables from census micro data)	√
(b) Published 100% census tabulations of age-sex-specific (and marital status-specific if possible) distributions of the entire population, and aggregated numbers of households		
**(2) Age-sex-specific standard schedules at the national level (can be used for projections at sub-national and county/city levels),** estimated by ProFamy R software DemoRates using commonly available data of demographic surveys or census micro file		
(a) Age-sex-(and marital-status if possible)-specific mortality rates;	√	√
(b) Age-specific fertility rates;	√	√
(c) Age-parity-specific occurrence/exposure (o/e) rates of marital and non-marital fertility;	√	
(d) Age-sex-specific occurrence/exposure (o/e) rates of marriages and divorces;	√	
(f) Age-sex-specific rates of international immigration and emigration, or age-sex-specific rates of international net-migration;	√	√
(g) Age-sex-specific rates of domestic in-migration and out-migration if the projections are for the sub-national regions;	√	√
(h) Age-sex-specific net rates of leaving the parental home, estimated based on two adjacent census micro data files and the intra-cohort iterative method (Coale, 1984, 1985; Stupp, 1988; Zeng et al., 1994), using the ProFamy software	√	
**(3) Demographic summary parameters for the nation or sub-national regions or counties in the baseline and selected future projection years**		
(a) Total fertility rates (TFR);	√	√
(b) Total marriage rates (TMR) and total divorce rates (TDR);	√	
(c) Sex-specific life expectancies at birth;	√	√
(d) Sex-specific general rates of in-migration and sex-specific general rates of out-migration;	√	√
(e) Mean age at births;	√	√
(f) Sex-specific mean ages at first marriage	√	

Notes: (1) The data categories of rural/urban (or race) are optional based on the actual demographic situation and data availability of the country or regions or county/city under study. If the categories of rural/urban (or race) are adopted in the application, all data listed in this table will need to be rural/urban (or race)-specific. (2) The total rates of marriages and divorces are defined as sums of the age-specific frequencies of marriages and divorces from the lowest age to the highest age at which events of marriages and divorces may occur, which is similar to the definition of total fertility rate

As Keyfitz (1972) pointed out, demographic projections based on trend extrapolation of each age-sex-specific rate could lead to an excessive concession to flexibility and easily produce erratic results. Accordingly, analysts worldwide tend to use fixed age-sex-specific standard schedules (Sect. (2) of Table 1) and concentrate on projecting future changing demographic summary parameters (Sect. (3) of Table 1) in population, household and living arrangement projections. Numerous studies have demonstrated that fixed age-sex-specific standard schedules and a few changing summary parameters offer an efficient and realistic approach for demographic projections (Brass, 1974; Coale et al., 1983). The theoretical foundation of this practice is that the changing demographic summary parameters are crucial to determine dynamics in levels and age patterns of age-specific rates that affect projections. At the same time, projection results typically are not highly sensitive to fixed age-sex-specific standard schedules. Thus, national age-sex-specific standard schedules can be readily employed for household and living arrangement projections at the sub-national and county/city levels using the ProFamy methods/software, as empirically tested in various studies (Smith et al., 2012; Zeng et al., 2013a, 2013b, 2013c, 2014), or even be used for projections or estimations in other countries with similar demographic patterns, as corroborated in Zeng et al. (2000).

Note that the age-sex-specific net rates of leaving the parental home, estimated based on two adjacent census micro data files and the intra-cohort iterative method (Coale, 1984, 1985; Stupp, 1988; Zeng et al., 1994), using the ProFamy software; the sex-specific mean ages at first marriage can be estimated easily using the conventionally available demographic survey datasets.

### Assessments of the ProFamy extended cohort-component method

Assessments validated the accuracy of projections using the ProFamy model for the United States and China from 1990 to 2000, namely, the forecast errors, measured by discrepancies between the projected values and the 2000 census observations, are reasonably small (Zeng et al., ). The test comparisons of the ProFamy projections from 1990 to 2000 with census counts in 2000 for each of the 50 U.S. states and DC showed that 63.0, 17.4, 12.9, and 6.7 percent of the absolute percentage errors were < 3.0%, 3.0–4.99%, 5.0–9.99% and ≧10.0%, respectively, among 306 pairs of comparisons of the main indices of household projections between forecasts and the census observations (Zeng et al., 2013a, 2013b, 2013c). Similar assessments for each of the 31 Chinese provinces show that the ProFamy methods/software works well. Tests of projections of households and living arrangements in 2000–2010 and comparing the results with the 2010 census counts for the six counties of Southern California validated the applications of ProFamy approach at county/city level (Feng et al., 2019).

As shown in Table 2, Another assessment compares average forecast errors between the ProFamy approach and the traditional headship-rate method, by projecting the number-of- bedrooms-specific housing demands from 1990 to 2000 and then comparing them with census counts in 2000 for each of the U.S. 50 states and DC. The results demonstrate that, as compared to the ProFamy approach, the headship-rate method produces much more serious forecast errors, because it projects household types only but not by sizes (Zeng et al., 2014).

**Table 2:** Mean forecasting errors of housing demand projections from 1990 to 2000 (compared to the 2000 census observations) for the 50 U.S. states and DC, produced by the ProFamy cohort-component approach and constant headship rates

	0–1 bedroom units			2-bedroom units			3-bedroom units			4-bedroom units		
	ProFamy	Headship constant	%Difference	ProFamy	Headship constant	%Difference	ProFamy	Headship constant	%Difference	ProFamy	Headship constant	%Difference
MALPE	− 6.25	− 18.67	198.7	2.51	5.01	99.6	1.38	4.3	211.6	1.15	− 3.23	− 380.9
MAPE	8.71	18.67	114.4	5.87	7.07	20.4	3.94	5.04	27.9	6.52	8.11	24.4
MEDAPE	8.11	18.52	128.4	4.86	6.66	37.0	3.10	3.51	13.2	5.19	7.96	53.4

Notes: (1) MALPE means “The Mean Algebraic Percent Error of forecasts”; MAPE means “The Mean Absolute Percent Error; MEDAPE means “Median Absolute Percent Error’. (2) The term of “0-bedroom housing unit” means that the bedroom is mixed with the living room

Source: Zeng et al. (2014; Table 4.8)

### Applications of the ProFamy for household and living arrangement projections

The ProFamy model and the user-friendly free software have been used to generate household and living arrangement projections in China, the United States, Canada, Austria, German, Brazil, Mexico, Sri Lanka, Pakistan, and Iran. These studies include: U.S. household and living arrangement projections by race (Jiang & O’Neill, 2007); implications of changes in U.S. households and living arrangements for the housing industry and policy-making (Smith et al., 2008, 2012); U.S. household projections and home-based energy demands and future carbon emissions (Dalton et al., 2008; O'Neill & Chen, 2002; O'Neill & Jiang, 2007); household automobile demands in the United States (Feng et al., 2011) and Austria (Prskawetz et al., 2004); fertility policy analyses, retirement ages and older adults care needs/costs studies in China (Feng et al., 2019; Zeng, 2007, 2011; Zeng et al., 2008, 2013a, 2013b, 2013c, 2014); German household and living arrangement projections (Hullen, 2000, 2003); household and living arrangement projections in Brazil (Tirza, 2017), Mexico (Landy, 2017); estimating the fiscal impacts of changing household structures in Sri Lanka (Gruen, 2020); households and residential energy demands projections in Pakistan (Hussain & Sadiq, 2020) and Hebei province of China (Zeng et al., 2021).

One could use the ProFamy methods/software to address the important research questions such as: how many children will live in single-parent (especially single-mother) households, and how many single-mothers will have to care for their 1, 2, 3 or more children with no spouse or partner present in the future years (Zeng et al., 2014)? Applications to address these research questions are practically feasible using the ProFamy methods/software, as it already includes children’s status of co-residence with two or one parent(s), and adults’ statuses of marital/union and number of co-residing children.

Populations and societies are aging quickly, and the age-specific prevalence of disability in daily living will increase sharply among older persons in almost all countries around the world. It is imperative to conduct analyses of and projections on households, older adults living arrangements, caregivers and home-based care needs/costs for older adults who have cognitive impairments (including dementia and Alzheimer’s disease) and/or physical impairments and are disabled in daily living; and the ProFamy extended cohort/component methods/software can make useful contributions to such studies (Zeng et al., 2013a, 2013b, 2013c, 2014: 167–188). It is also very useful to apply the ProFamy model/software for addressing interesting research questions on, in the future years, how many older adults will have 0, 1, 2, 3 or more surviving adult children, and how many young and middle-age adults will have to take care of surviving old parent(s) plus 0, 1, 2, 3 or more young kids, namely, the so-called “over-load” middle-generation (Zeng et al., 2014: 330).

The ProFamy model/software has also been used to produce household projections at sub-national and county/city levels for socioeconomic planning in the United States (Feng et al., 2020; Smith et al., 2012; Zeng et al., 2013a, 2013b, 2013c), Germany (Hullen, 2003), Brazil (Tirza, 2017), Iran (Bagi, 2018), Mainland, Macau Special District, Taiwan and other provinces of China (Jiang & Kuijsten, 1999a, 1999b; Wang & Qiao, 2018; Yang & Zeng, 2000; Zeng et al., 2008). A notable example is that the local governmental office employed ProFamy methods/software, the U.S. national race-age-sex-specific standard schedules and the demographic summary parameters at the county level to successfully project households and living arrangements for six counties in Southern California since 2009, with projections renewed every two years. The six counties’ governments have effectively used these detailed biennial projections for their socio-economic planning, budget allocations, and policy analyses on housing, traffic, energy demands, older adults care and other home-base social services (Feng et al., 2020).

Up to Nov. 6, 2024, according to incomplete statistics, there are 287 registered users (excluding their co-authors, students and other group members) from 39 countries, the United Nations Fund for Population Activities (UNFPA), and the World Bank downloaded and used the ProFamy extended cohort-component methods and the associated free software (http://www.profamy.com.cn/#/home) to do research on household and living arrangement projections for healthy aging, sustainable development and informed decision-making research.

### Using ProFamy user-friendly software BasePop subroutine for empirical analyses on household dynamics based on census datasets

The BasePop subroutine of ProFamy free software can also be used to utilize micro censuses datasets available to the users (or publicly available at IPUMS database of University of Minnesota; https://www.ipums.org/mission-purpose) and the officially published census 100% summary cross-tabulations to produce not only proportions but also 100% absolute numbers of households by types and sizes and older adults, young & middle-ages and children by various living arrangements.

Governmental socioeconomic planning and private business market analyses need not only detailed proportion distributions but also absolute numbers of households by types and sizes living arrangements of older adults, children, youth, and middle-aged adults. In some circumstances, the information of dynamic changes in absolute numbers is more useful than that of proportions in socioeconomic planning and market analyses. For example, the absolute numbers of Chinese people aged 80 + living alone (who need care services) increased by 233.2 percent from 1990 to 2010. In contrast, the increase in the proportion of the oldest-old living alone among total population was only 21.8 percent in the same period.

One could apply the user-friendly BasePop subroutine of the ProFamy model and software to estimate both proportions and detailed absolute numbers of 100 percent population distributions of households by types/sizes and living arrangements of older adults, children, youth, and middle-aged adults by age, sex and rural–urban residence (or race) in the census year, while ensuring internal consistencies and accuracy. Such applications of the BasePop subroutine of the ProFamy software not only produces a baseline for the household and living arrangement projections, but also facilitates the analyses for those who are interested in dynamics of households and living arrangements in the past years by analysing 2 or 3 or more censuses micro datasets available to them (or publicly available at IPUMS database of University of Minnesota).

### Using ProFamy user-friendly R software DemoRates to estimate the age-sex-specific standard schedules and total rates of the demographic rates

As demonstrated in Table 1, the majority of the input data needed for household and living arrangement projections using the ProFamy methods/software are the same as those required for the population projections (including PPPs). The additional work of data preparation for household and living arrangement projections using the ProFamy extended cohort-component methods/software is to estimate rural/urban (or race)-age-specific standard schedules of o/e rates of marital and non-marital fertility by parity, marriages and divorces (abbreviated as “sex-age-status-specific standard schedules of o/e rates” hereafter). Note that lack of sex-age-status-specific standard schedules of o/e rates is the main barrier of conducting detailed and useful projections of households and living arrangements. However, the needed sex-age-status-specific standard schedules, total marriage rates and total divorce rates can in fact be estimated using the commonly available data collected in the ordinary demographic surveys in almost all countries (Zeng et al., 2012: 214–215). The reason why they are lacking is that it is not easy to adequately estimate and smooth schedules of o/e rates using the standard statistical software.

To assist researchers in estimating and smoothing schedules of o/e rates, other age-status-specific demographic rates/frequencies, total marriage rates and total divorce rates for household and living arrangement projections and other demographic research, we developed the user-friendly R package DemoRates, as part of the ProFamy free software package (http://www.profamy.com.cn/#/home).

## ProFamy approach provides an adequate and highly feasible modelling framework to do probabilistic household and living arrangement projections (PHPs) based upon PPPs of UNPD

The current version of ProFamy methods follows the classic deterministic projection approach which has three inherent limitations. First, it presents uncertainty of the projections by providing high, medium and low scenarios based on experts' educated guesses or speculations about the combinations of different demographic parameters in the future, but such subjective “what if…” scenarios are not designed to provide a range of the plausible projections of future trends (Lutz & Kc, 2010). Second, it is not statistically based on the demographic parameters’ past trajectories that may affect future trends. Third, it does not provide probabilistic predictive intervals for the projection outcomes, namely, it does not provide the L80 and U80 (which are the low and high boundaries of the 80% predictive interval of the projected outcome), and the L95 and U95 (which are the low and high boundaries of the 95% predictive interval of the projected outcome). This is why we do need to substantially extend the ProFamy cohort-component methods/software from deterministic to the new PHPs methods/software in order to overcome the three inherent limitations of the classic deterministic projections as outlined above, for the users of PHPs to more effectively plan interventions.

As shown in Table 1, while most of the data needed are the same as those used for population projections (including UNPD PPPs), using the ProFamy methods/software for simultaneously projecting households, living arrangements and population age/sex distributions mainly need the following additionally sex-age-specific demographic rates:

1. Sex-age-specific occurrence/exposure (o/e) rates and total rates of marriages and divorces;
2. Age-parity-specific o/e rates of marital and non-marital fertility;

The demographic rates of (1) and (2) can be estimated using the ordinary demographic survey datasets, the event history analysis method, and the ProFamy user-friendly R package DemoRates.

As discussed in Sect. 3.2, similar to the population projections (including the UNPD PPPs), the ProFamy extended cohort-component model uses fixed age-sex-specific demographic standard schedules (panel (2) of Table 1) and concentrate on projecting the several changing summary parameters (panel (3) of Table 1) to formulate various scenarios of household and living arrangement projections. Thus, the ProFamy extended cohort-component method provides an adequate and highly feasible modelling framework to extend the probabilistic population projections (PPPs), which does not include household components. We plan to develop new methods/software of probabilistic household and living arrangement projections (PHPs) with uncertainty intervals. We also plan to develop the international database that can be used by the international researchers for the PHPs in the less developed countries in which the demographic data sources are poor.

## Conclusions, recommendations, progress, and perspectives

As reviewed in Sects. 2 and 3, the ProFamy extended cohort-component method for household and living arrangements projections uses the commonly available demographic data and can be employed to investigate how dynamics of household types/sizes, living arrangements, and life courses of individuals are affected by changes in demographic rates of marriages, divorces, fertility, migration, and mortality. One could use the ProFamy user-friendly R package “DemoRates” and the commonly available demographic surveys and census datasets to relatively easily estimate rural/urban (or race)-age-sex-specific standard schedules of occurrence/exposure (o/e) rates of marriages, divorces, marital, and non-marital fertility by parity and migration, as well as the total rates of marriages, divorces, fertility by parity and migration. Clearly, such research would effectively strengthen household and living arrangement projections for informed decision making, and also to enhance other types of demographic research on family and life course analyses.

For the sound policy-making and socioeconomic planning, we recommend using the ProFamy methods/software to address research questions such as: how many children will live in single-parent (especially single-mother) households, and how many single mothers will have to care for their 1, 2, 3 or more children with no spouse or partner present in the future years? We also believe that it is very important to strengthen analyses and projections on households, older adults living arrangements, caregivers and home-based care needs/costs for elders who are disabled in daily living, employing the ProFamy methods/software. We recommend employing the ProFamy methods/software to conduct household types/sizes and home-based energy and housing demands projections, which are crucially important for strategic planning of disabled older adults friendly housing, home-based energy demands, environment protections and sustainable development.

Prior studies have found a significant and negative correlation between Total fertility rates (TFR(t)) and life expectancies at birth (e0(t)), based on either country-specific time series data (Jannat, 2022; Jafrin et al., 2021; Onwube et al., 2021) or cross-sectional data from multiple countries (Girum et al., 2018; Kalita, 2019; Mondal et al., 2015). Our pilot research found that complete random-combinations of TFR(t) and e0(t) would result in substantially high percentages of improbable combinations in probabilistic population projections (PPPs) for developing and developed countries, respectively, namely, high (or low) TFR(t) combined with high (or low) female e0(t) (Li et al., 2024). Thus, we propose optimized random-combinations of probabilistically projected TFR(t) and e0(t) for PPPs. As illustrative applications, we conducted PPPs for 11 developing countries (Brazil, China, Indonesia, Madagascar, Pakistan, Philippines, Saudi Arabia, Singapore, Sri Lanka, Thailand, Viet Nam), and 6 developed countries (Canada, France, Italy, Japan, the United Kingdom, the United States), using our proposed optimized random-combinations of probabilistically projected TFR(t) and e0(t) (Li et al., 2024). We found that optimized random-combinations largely reduce percentages of improbable combinations of TFR(t) and e0(t) and substantially narrow the prediction intervals width compared to complete random-combinations in both developing countries and developed countries (Li et al., 2024). This is important in a real-world practical sense since it would substantially improve the accuracy of PPPs, which are useful for socioeconomic planning.

As reported in Zeng et al. (2024), we have made substantial progress in our ongoing international collaborative project on PHPs research. We have successfully estimated the time series of total marriage rates (TMR(t)) and total divorce rates (TDR(t)) in 1950–2020 in China and in 1960–2020 in United States in 1960–2020 estimated by the BayesRates R package which is part of the ProFamy DemoRates software. Based on these estimates of time series of TMR(t) and TDR(t), we conducted probabilistic projections of the trajectories of TMR(t) and TDR(t) in 2021–2100 for China and United States using the BayesProj R package (Zhang et al., 2024) for our ongoing PHPs research. This is of course not enough. In addition to the two case studies of China and United States as outlined above, we plan to estimate the time series of TMR(t) and TDR(t) in the past decades, and based on these estimates, we will conduct probabilistic projections of the trajectories of TMR(t) and TDR(t) in 2021–2100 for the 11 countries Brazil, Indonesia, Iran, Malaysia, Pakistan, Philippines, Saudi Arabia, Singapore, Sri Lanka, Thailand, United Kingdom, and Viet Nam, whose scholars have signed collaboration agreements with us and they are actively participating in our ongoing international collaborative research project on PHPs.

We will be in collaboration with UNPD to conduct the indirect estimations (similar to the indirect estimations using the UN model life tables) of the needed demographic rates for the less developed countries with poor demographic data source. To reach this goal, we will apply the Bayesian model (which is good to deal with poor data) and the DemoRates R package, and the conventionally available demographic survey datasets to estimate the time series the trajectories of TMR(t) and TDR(t) in the past years for each of the countries. We will develop user-friendly software ProFamy-PHPs and international database (both PHPs software and database are free of charge), so that researchers can relatively easily conduct probabilistic household and living arrangement projections using the commonly available demographic data as inputs.

As discussed in the Sect. 4, the ProFamy extended cohort-component method provides an adequate and highly feasible modelling framework for research to substantially extend the United Nations Population Division (UNPD) probabilistic population projections (PPPs), which does not include household components, to innovative methods/software of probabilistic household and living arrangement projections (PHPs) with uncertainty intervals. We will closely collaborate with the experts from UNPD and other institutions who do the PPPs. Our aim is to collaboratively extend PPPs to PHPs in which the population projection outcomes are consistent with the PPPs released by UNPD, while PHPs produce a lot of additional outcomes of probabilistically projected households and living arrangements in 2021–2100 for studies on healthy aging, sustainable development, and the informed decision-making.

## Figures and Tables

**Fig. A1.**
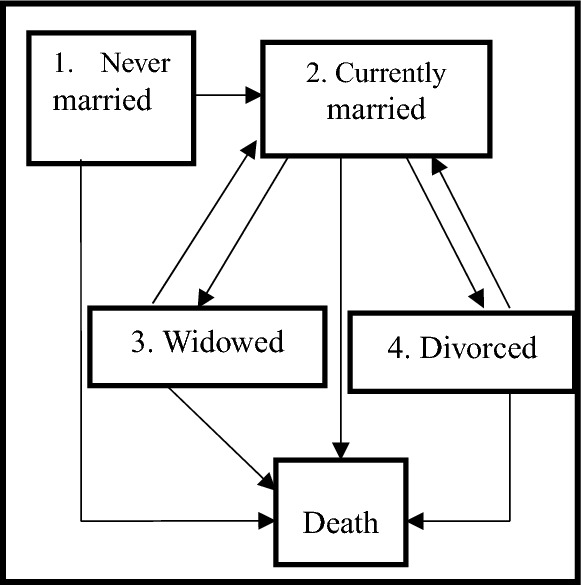
Transitions of 4 marital statuses; Source: Figs. A1 and A2 of Zeng et al., (2014), pages 20–21

**Fig. A2.**
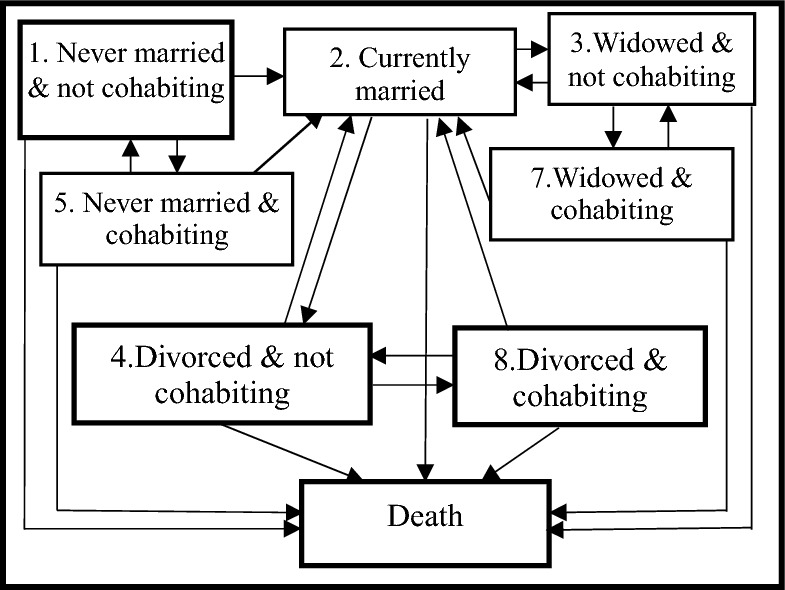
Transitions of 7 marital/union statuses; Source: Figs. A1 and A2 of Zeng et al., (2014), pages 20–21

## Data Availability

Data availability is not applicable to this article as no new data were created or analyzed in this study.

## Appendix: Decompose the probabilistically projected 4 marital statuses into 7 marital/union statuses

Using the ProFamy methods/software to do the projections of households and living arrangements, users can choose either the multistate transitional model of four marital statuses (never married, currently married, widowed and divorced; ref. Fig. A1) or seven marital/union statuses (never married & not-cohabiting, currently married, widowed & not-cohabiting, divorced & not-cohabiting, never married & cohabiting, widowed & cohabiting, divorced & cohabiting; Zeng et al., 2014). However, if the seven-marital/union-status-transitional model is adopted in the PHPs, one would need to estimate the time series and probabilistically project the summary measures of thirteen marital/union status transitions, in addition to estimate thirteen sets of rural/urban (or race)-sex-age-specific standard schedules (ref. Fig. A2), namely;

1. transitions from never-married & not-cohabiting to currently married,
2. transitions from never-married & not-cohabiting to never-married & cohabiting;
3. transitions from never-married & cohabiting to currently married;
4. transitions from never-married & cohabiting to never-married & not-cohabiting;
5. transitions from currently married to divorced & not-cohabiting;
6. transitions from widowed & not-cohabiting to currently married;
7. transitions from widowed & not-cohabiting to widowed & cohabiting;
8. transitions from widowed & cohabiting to currently married;
9. transitions from widowed & cohabiting to widowed & not-cohabiting;
10. transitions from divorced & not-cohabiting to currently married;
11. transitions from divorced & not-cohabiting to divorced & cohabiting;
12. transitions from divorced & cohabiting to currently married;
13. transitions from divorced & cohabiting to divorced & not-cohabiting.

This is too much data-demanding and not feasible, since most countries worldwide do not have time series data on cohabitation statuses transitions, given the fact that cohabitations became popular in rather recent years. Thus, we follow the Sullivan proportional distribution approach to project cohabitation statuses in the future years in the PHPs. More specifically, we first employ the extended ProFamy multistate cohort-component model to probabilistic project households and living arrangements with four marital statuses (m = 1, never married; m = 2, currently married, m = 3: widowed; m = 4, divorced), and then decompose the three not-currently married statuses into cohabiting and not-cohabiting, respectively. In this way, we only need to estimate the age-specific standard schedules, estimate the time series and probabilistically project the summary measures of four marital statuses transitions, as follows:

1. transitions from never-married (m = 1) to currently married (m = 2);
2. transitions from currently married (m = 2) to divorced (m = 4);
3. transitions from widowed (m = 3) to currently married (m = 2);
4. transitions from divorce (m = 4) to currently married (m = 2).

After the probabilistic household and living arrangement projections are produced, we decompose the four marital statuses into seven marital/union statuses (m = 1, never married & not-cohabiting; m = 2, currently married; m = 3, widowed & not-cohabiting; m = 4, divorced & not-cohabiting; m = 5, never married & cohabiting; m = 6, widowed & cohabiting; m = 7, divorced & cohabiting), using the simple formulas presented in the Appendix. In this way, we avoid the very complicated works and data-intensive task of estimating age-specific standard schedules, estimating time series of and probabilistically projecting summary measures of transitions among seven marital/union statuses, as shown in Fig. A2.

Denote N4(x,m,c,k,s,r,t) as the probabilistically projected numbers of persons with age x, marital status m (m = 1: never married; m = 2: currently married; m = 3: widowed; m = 4: divorced), co-residence with children status c, co-residence with parent(s) status k, sex s, and rural/urban (or race) status r in the future years t; N7(x,m,c,k,s,r,t) as the decomposed and estimated numbers of persons with age x, sex s, marital/union status m (m = 1: never married & not-cohabiting; m = 2: currently married; m = 3: widowed & not-cohabiting; m = 4: divorced & not-cohabiting; m = 5: never married & cohabiting; m = 6: widowed & cohabiting; m = 7: divorced & cohabiting), c, k, and r statuses in year t; N7(x,2,c,k,s,r,t) = N4(x,2,c,k,s,r,t).

Denote Pc(x,m,s,r,t) as the projected proportions of persons who are cohabiting among those with age x, marital status m (m = 1,3,4), sex s, and rural/urban (or race) r in year t; AvPc(m,s,r,T1) as the weighted average proportions (over ages) of persons who are cohabiting among those with marital status m (m = 1,3,4), sex s, and rural/urban (or race) r in year T1 (T1 is the most recent census year), based on the observed Pc(x,m,s,r,T1), which are estimated using the census data (or the recent survey data if the census does not collect cohabitation data). We estimate the AvPc(m,s,r,T1) using formula (A1), in which the weights are ${\text{Pc}}\left( {{\text{x}},{\text{m}},{\text{s}},{\text{r}},{\text{T1}}} \right) \, /\sum\nolimits_{x = 15}^{\beta + } {Pc(x,m,s,r,T1)}.$

A1 $$  {\text{AvPc}}\left( {{\text{m}},{\text{s}},{\text{r}},{\text{T1}}} \right) \, = \sum\limits_{x = 15}^{\beta + } {\left[ {Pc(x,m,s,r,T1)*\frac{Pc(x,m,s,r,T1)}{{\sum\limits_{x = 15}^{\beta + } {Pc(x,m,s,r,T1)} }}} \right]} ;{\text{ m}} = {1},{3},{4}$$

Pc(x,m,s,r,t) in the future years t (t > T1) are projected based on the observed Pc(x,m,s,r,T1) and AvPc(m,s,r,T1) in year T1 as well as the projected (or assumed) AvPc(m,s,r,t) in future year t, using the formula (A2); one may project (or assume) that AvPc(m,s,r,t) increase or decrease in the future years t based on observed trends in the recent past years.

A2 $$ {\text{Pc}}\left( {{\text{x}},{\text{m}},{\text{s}},{\text{r}},{\text{t}}} \right) \, = {\text{ Pc}}\left( {{\text{x}},{\text{m}},{\text{s}},{\text{r}},{\text{T1}}} \right) \, *\frac{{AvPc\left( {m,s,r,t} \right)}}{{AvPc\left( {m,s,r,T1} \right)}};{\text{ i}} = {1},{3},{4};{\text{ t}} > {\text{T1}} $$

We multiply the N4(x,m,c,k,s,r,t) by Pc(x,m,s,r,t) (m = 1, 3, 4) to project numbers of persons who are cohabiting or not-cohabiting in future years t, using the following simple formulas (A3 ~ A8):

A3 $$ {\text{N7}}\left( {{\text{x}},{5},{\text{c}},{\text{k}},{\text{s}},{\text{r}},{\text{t}}} \right) \, = {\text{ N4}}\left( {{\text{x}},{1},{\text{c}},{\text{k}},{\text{s}},{\text{r}},{\text{t}}} \right)*{\text{Pc}}\left( {{\text{x}},{1},{\text{s}},{\text{r}},{\text{t}}} \right); $$

A4 $$ {\text{N7}}\left( {{\text{x}},{6},{\text{c}},{\text{k}},{\text{s}},{\text{r}},{\text{t}}} \right) \, = {\text{ N4}}\left( {{\text{x}},{3},{\text{c}},{\text{k}},{\text{s}},{\text{r}},{\text{t}}} \right)*{\text{Pc}}\left( {{\text{x}},{3},{\text{s}},{\text{r}},{\text{t}}} \right); $$

A5 $$ {\text{N7}}\left( {{\text{x}},{7},{\text{c}},{\text{k}},{\text{s}},{\text{r}},{\text{t}}} \right) \, = {\text{ N4}}\left( {{\text{x}},{4},{\text{c}},{\text{k}},{\text{s}},{\text{r}},{\text{t}}} \right)*{\text{Pc}}\left( {{\text{x}},{4},{\text{s}},{\text{r}},{\text{t}}} \right); $$

A6 $$ {\text{N7}}\left( {{\text{x}},{1},{\text{c}},{\text{k}},{\text{s}},{\text{r}},{\text{t}}} \right) \, = {\text{ N4}}\left( {{\text{x}},{1},{\text{c}},{\text{k}},{\text{s}},{\text{r}},{\text{t}}} \right) \, - {\text{ N7}}\left( {{\text{x}},{5},{\text{c}},{\text{k}},{\text{s}},{\text{r}},{\text{t}}} \right); $$

A7 $$ {\text{N7}}\left( {{\text{x}},{3},{\text{c}},{\text{k}},{\text{s}},{\text{r}},{\text{t}}} \right) \, = {\text{ N4}}\left( {{\text{x}},{3},{\text{c}},{\text{k}},{\text{s}},{\text{r}},{\text{t}}} \right) \, - {\text{ N7}}\left( {{\text{x}},{6},{\text{c}},{\text{k}},{\text{s}},{\text{r}},{\text{t}}} \right); $$

A8 $$ {\text{N7}}\left( {{\text{x}},{4},{\text{c}},{\text{k}},{\text{s}},{\text{r}},{\text{t}}} \right) \, = {\text{ N4}}\left( {{\text{x}},{4},{\text{c}},{\text{k}},{\text{s}},{\text{r}},{\text{t}}} \right) \, - {\text{ N7}}\left( {{\text{x}},{7},{\text{c}},{\text{k}},{\text{s}},{\text{r}},{\text{t}}} \right); $$

## References

[CR5] Bagi, M. (2018). Household and living arrangement projections for Iran. PhD thesis research, Department of Demography, Faculty of Social Sciences, University of Tehran, Tehran, Iran.

[CR6] Bardazzi, R., & Pazienza, M. G. (2017). Switch off the light, please! Energy use, aging population and consumption habits. *Energy Economics,**65*, 161–171.

[CR7] Bell, M., Cooper, J. (1990). Household forecasting: Replacing the headship rate model. Paper Presented at the Fifth National Conference, Australian Population Association, Melbourne.

[CR9] Bongaarts, J. (1987). The Projection of family composition over the life course with family status life tables. In J. Bongaarts (Ed.), *Family Demography: Methods and Applications* (pp. 189–212). Clarendon Press.

[CR10] Bradbury, M., Peterson, M. N., & Liu, J. (2014). Long-term dynamics of household size and their environmental implications. *Population and Environment*. 10.1007/s11111-014-0203-6

[CR11] Brass, W. (1974). Perspectives in population prediction: Illustrated by the statistics of England and Wales. *Journal of the Royal Statistical Society,**137*(Series A), 532–583. 10.2307/2344713

[CR12] Casey, T., Maldonado, L. (2012). Worst off–single-parent families in the United States. A cross-national comparison of single parenthood in the US and Sixteen Other High-Income Countries. New York: Legal Momentum, the Women’s Legal Defense and Education Fund.

[CR13] Chen, C.-F., Xu, X., Adua, L., Briggs, M., & Nelson, H. (2022). Exploring the factors that influence energy use intensity across low-, middle-, and high-income households in the United States. *Energy Policy,**168*, 113071.

[CR14] Christiansen, S. G., & Keilman, N. (2013). Probabilistic household forecasts based on register data—The case of Denmark and Finland. *Demographic Research,**28*, 1263–1302. 10.4054/DemRes.2013.28.43

[CR15] Coale, A. J. (1984). Life table construction on the basis of two enumerations of a closed population. *Population Index,**50*, 193–213. 10.2307/2736754 12339444

[CR16] Coale, A. J. (1985). An extension and simplification of a new synthesis of age structure and growth. *Asian and Pacific Forum,**12*, 5–8. 12267195

[CR17] Coale, A. J., Demeny, P., & Vaughn, B. (1983). *Regional model life tables and stable populations*. Academic Press.

[CR18] Dalton, M., O’Neill, B., Prskawetz, A., et al. (2008). Population aging and future carbon emissions in the United States. *Energy Economics,**30*, 642–675. 10.1016/j.eneco.2006.07.002

[CR19] Dubois, G., Sovacool, B., Aall, C., et al. (2019). It starts at home? Climate policies targeting household consumption and behavioral decisions are key to low-carbon futures. *Energy Research & Social Science,**52*, 144–158. 10.1016/j.erss.2019.02.001

[CR20] Feng, Q., Choi, S., Wang, Z., & Zeng, Y. (2020). Forecast households at the county level: An application of the ProFamy extended cohort-component method in six counties of Southern California, 2010 to 2040. *Population Research and Policy Review,**39*, 253–281. 10.1007/s11113-019-09531-4

[CR21] Feng, Q., Wang, Z., Gu, D., & Zeng, Y. (2011). Household vehicle consumption forecasts in the United States, 2000 to 2025. *International Journal of Market Research,**53*(5), 593–618. 10.2501/IJMR-53-5-593-618

[CR22] Feng, Q., Yeung, J. W., Wang, Z., & Zeng, Y. (2019). Age of retirement and human capital in an aging China, 2015–2050. *European Journal of Population,**35*(1), 29–62. 10.1007/s10680-018-9467-3 30976267 10.1007/s10680-018-9467-3 PMC6357252

[CR23] Fox, J. (2002). *Cox proportional-hazards regression for survival data*. An R and S-PLUS Companion to Applied Regression. https://socialsciences.mcmaster.ca/jfox/Books/Companion-2E/appendix/Appendix-Cox-Regression.pdf

[CR24] Francesca, C., Ana, L. N., Jérôme, M., et al. (2011). *OECD health policy studies help wanted? Providing and paying for long-term care: Providing and paying for long-term care*. OECD Publishing.

[CR900] Girum, T., Muktar, E., & Shegaze, M. (2018). Determinants of life expectancy in low and medium human development index countries. *Medical Studies/Studia Medyczne,**34*(3), 218–225. 10.5114/ms.2018.78685

[CR25] Gruen, C. (2020). Estimating the fiscal impacts of changing household structures in Sri Lanka using ProFamy Method/software; Part of a World Bank study on the impact of ageing in Sri Lanka.

[CR500] Grundy, E. (2013). Opening keynote speech at the households and living arrangement projections: new method. In: *Software and applications held at the 27th General conference of international union for scientific population studied (IUSSP), August 30 (the entire conference was held in August 26–31)*, vol 30. Busan, Korea, p 2013

[CR26] Gu, D., Feng, Q, Wang, Z. & Zeng, Y. (2015). Recommendation to consider the crucial impacts of changes in smaller household size and its structure on sustainable development goals. United Nations Sustainable Development Knowledge Platform, Scientific Briefs, 2015 (online), https://sustainabledevelopment.un.org/topics/science/crowdsourcedbriefs.

[CR28] Hammel, E. A. (2005). Demographic dynamics of kinship in anthropological populations. *Proceedings of the National Academy of Science,**102*(6), 2248–2253. 10.1073/pnas.0409762102 10.1073/pnas.0409762102 PMC548587 15677714

[CR29] Hullen, G. (2000). Projections of living arrangement, household and family structures using ProFamy. Warschau, Deutsch-polnisch-ungarisches Demographentreffen.

[CR30] Hullen, G. (2003). Living arrangements and households: Methods and results of demographic projection. A book (Reader) Published by the German Federal Institute for Population Research (BIB). http://www.gert-hullen.privat.t-online.de/manuskripte/materialien_hu_29072003.pdf.

[CR31] Hussain, S., Sadiq, M. (2020). Households, living arrangements, and residential energy demands projections in Pakistan; ongoing research project of Population Council Office in Pakistan.

[CR32] IEA (International Energy Agency). (2004). *30 Years of energy use in IEA countries*. International Energy Agency.

[CR678] Jafrin, N., Masud, M. M., Seif, A. N. M., Mahi, M., & Khanam, M. (2021). A panel data estimation of the determinants of life expectancy in selected SAARC countries. *Operations Research and Decisions,**31*(4), 69–87. 10.37190/ord210404

[CR890] Jannat, Z. (2022). The dynamics of female life expectancy and total fertility rate in Bangladesh: a time-series analysis. *International Journal of New Economics and Social Sciences IJONESS,**15*(1), 9–20. 10.5281/zenodo.7111914

[CR33] Jiang, L., Kuijsten, A. (1999b). Household projections for two regions of China. Paper Presented at the European Population Conference, the Hague, The Netherlands, August 30–September 3, 1999.

[CR34] Jiang, L., Kuijsten, A. (1999a). Effects of changing households on environment - case studies in two regions of China. Paper Presented at Workshop on Population and Environment: Modeling and Simulating This Complex Interaction, Organized by Max Planck Institute for Demographic Research at Rostock, Germany, August 12–13, 1999.

[CR35] Jiang, L., & O’Neill, B. C. (2007). Impacts of demographic trends on US household size and structure. *Population and Development Review,**33*(3), 567–591. 10.1111/j.1728-4457.2007.00186.x

[CR789] Kalita, U. (2019). An econometric investigation into variations in life expectancy at birth across countries. *International Journal of Science and Research (IJSR),* 1832–1836. 10.21275/ART2020679

[CR36] Keilman, N. (1985). Nuptiality models and the two-sex problem in national population forecasts. *European Journal of Population,**1*(2/3), 207–235. 12340530 10.1007/BF01796933

[CR37] Keilman, N. (1988). Dynamic household models. In N. Keilman, A. Kuijsten, & A. Vossen (Eds.), *Modelling household formation and dissolution* (pp. 123–138). Clarendon Press.

[CR38] Keilman, N. (2003). The threat of small households. *Nature,**421*, 489–490. 12556874 10.1038/421489a

[CR39] Keilman, N. (2018). Family projection methods: A review. In R. Schoen (Ed.), *Analytical family demography. *Springer Publisher.

[CR40] Keyfitz, N. (1972). On future population. *Journal of American Statistical Association,**67*, 347–363. 10.1080/01621459.1972.10482386 12309295

[CR41] Korotayev, A., Malkov, S., & Musieva, J., et al. (2023). Demography: Toward optimization of demographic processes. In V. Sadovnichy (Ed.), *Reconsidering the limits to growth, world-systems evolution and global futures* (pp. 97–116). Springer Nature. 10.1007/978-3-031-34999-7_6

[CR42] Lagakos, S. W. (1981). The graphical evaluation of explanatory variables in proportional hazard regression models. *Biometrika,**68*(1), 93–98.

[CR44] Land, K. C., & Rogers, A. (1982). *Multidimensional mathematical demography*. Academic Press.

[CR45] Landy S. (2017). Unfolding consumption: An application of household projections to estimate environmental implications of energy consumption. Paper Presented at the International Union for Scientific Studies of Population (IUSSP) 28th General Conference Side-meeting on “Households/Population Projections and Sustainable Development”, October 30, 2017, Cape Town, South Africa.

[CR46] Li, M., Zhu, S., Wang, Z., Feng, Q., Chao, F., Zhang, J., George, L., Grundy, E., Michael, M., Lutz, M., Dobra, A., Land, K., Yi, Z. (2024). Optimized Random-combinations of TMR(t), TDR(t), TFR(t) and Female e0(t) for Probabilistic Household and living arrangement projections. Forthcoming in *Population Research and Policy Review*.

[CR800] Lutz, W., & Kc, S. (2010). Dimensions of global population projections: what do we know about future population trends and structures? *Philosophical Transactions of the Royal Society B: Biological Sciences,**365*(1554), 2779–2791. 10.1098/rstb.2010.0133 10.1098/rstb.2010.0133 PMC2935115 20713384

[CR47] Maldonado, L. C., & Nieuwenhuis, R. (2015). Family policies and single parent poverty in 18 OECD Countries, 1978–2008. *Community, Work & Family,**18*(4), 395–415. 10.1080/13668803.2015.1080661

[CR48] Mason, A., & Racelis, R. (1992). A comparison of four methods for projecting households. *International Journal of Forecasting,**8*, 509–527. 12157869 10.1016/0169-2070(92)90061-d

[CR8000] Mondal, M. N. I., & Shitan, M. (2015). Author’s response regarding the relative importance of demographic, socioeconomic and health factors on life expectancy in low-and lower-middle-income countries. *Journal of Epidemiology,**25*(6), 460. 10.2188/jea.JE20130059 25986157 10.2188/jea.JE20150046 PMC4444502

[CR49] Murphy, M. (1991). Modelling households. A synthesis. In M. J. Murphy & J. Hobcraft (Eds.), *Population research in Britain, a supplement to population studies* (Vol. 45, pp. 151–176). London: Population Investigation Committee, London School of Economics.

[CR50] O'Neill, B. C., Jiang, L. (2007). Projecting U.S. household changes with a new household model. IIASA Interim Report. IIASA, Laxenburg, Austria: IR-07–017.

[CR51] O’Neill, B. C., & Chen, B. S. (2002). Demographic determinants of household energy use in the United States. *Population and Development Review,**28*(S), 53–88.

[CR52] Park, H. C., & Heo, E. (2007). The direct and indirect household energy requirements in the Republic of Korea from 1980 to 2000—An input–output analysis. *Energy Policy,**35*, 2839–2851. 10.1016/j.enpol.2006.10.002

[CR53] Pawlak, J., Faghih Imani, A., & Sivakumar, A. (2021). How do household activities drive electricity demand? Applying activity-based modelling in the context of the United Kingdom. *Energy Research & Social Science,**82*, 102318.

[CR54] Prskawetz, A., Jiang, L., & O’Neill, B. C. (2004). Demographic composition and projections of car use in Austria. *Vienna yearbook of population research* (pp. 274–326). Austrian Academy of Sciences Press.

[CR55] Smith, S. K., Rayer, S., & Smith, E. A. (2008). Aging and disability: Implications for the housing industry and housing policy in the United States. *Journal of the American Planning Association,**74*, 289–306. 10.1080/01944360802197132

[CR56] Smith, S. K., Rayer, S., Smith, E. A., Wang, Z., & Zeng, Y. (2012). Population aging, disability and housing accessibility: Implications for sub-national areas in the United States. *Housing Studies,**27*(2), 252–266. 10.1080/02673037.2012.649468

[CR57] Spicer, K., Diamond, I., & Bhrolchain, M. N. (1992). Into the twenty-first century with British households. *International Journal of Forecasting,**8*, 529–539. 10.1016/0169-2070(92)90062-E 12157870 10.1016/0169-2070(92)90062-e

[CR58] Stupp, P. W. (1988). A general procedure for estimating intercensal age schedules. *Population Index,**54*, 209–234. 12341806

[CR59] Swanson, D. A., & Pol, L.G. (2009). In Y. Zeng (Eds.), *Encyclopedia of life support systems vol 2 demography,* (EOLSS) (www.eolss.net), Coordinated by the UNESCO-EOLSS Committee. Oxford, UK: EOLSS Publishers Co. Ltd.

[CR60] Thorve, S., Baek, Y.Y., Swarup, S., Mortveit, H., Marathe, A., Vullikanti A. & Marathe, M. (2023). High resolution synthetic residential energy use profiles for the United States. Scientific Data, Open Data Descriptor; www.nature.com/scientificdata. 10.1038/s41597-022-01914-1 PMC9902520 36746951

[CR61] Tirza, A. (2017). Elderly population and their living arrangements in the most developed state of Brazil. Paper Presented at the International Union for Scientific Studies of Population (IUSSP) 28th General Conference Side-meeting on “Households/Population Projections and Sustainable Development”, October 30, 2017, Cape Town, South Africa.

[CR63] van Imhoff, E., & Keilman, N. (1992). *LIPRO 2.0: An application of a dynamic demographic projection model to household structure in the Netherlands*. Amsterdam: Swets & Zeithinger.

[CR64] van Imhoff, E., Kuijsten, A., Hooimeiger, P., & van Wissen, L. (1995). Epilogue. In E. Imhoff, A. Kuijsten, P. Hooimeiger, & L. van Wissen (Eds.), *Household demography and household modeling* (pp. 345–351). Plenum.

[CR65] Wachter, K. W. (1987). Microsimulation of household cycles. In E. Bongaarts, T. K. Burch, & K. W. Wachter (Eds.), *Family demography: Methods and applications* (pp. 215–227). Clarendon Press.

[CR66] Wang, Z., & Qiao, X. (2018). Housing demands forecasting and policy analysis in Macau Special District of China. *Macau Newspaper,**9*, 2018. in Chinese.

[CR67] Willekens, F. (2009). Continuous-time microsimulation in longitudinal analysis. In A. Zaidi, A. Harding, & P. Williamson (Eds.), *New frontiers in microsimulation modelling* (pp. 413–436). Ashgate Publishing Ltd.

[CR68] Willekens, F. (2010). Family and household demography. demographyIn Y. Zeng (Ed.), *Encyclopedia of life support systems* (Vol. 2, pp. 86–112). UNESCO in partnership with EOLSS Publishers.

[CR69] Willekens, F., Shah, I., Shah, J. M., & Ramachandran, P. (1982). Multistate analysis of marital status life tables: Theory and application. *Population Studies,**36*, 129–144.

[CR70] Wilson, T. (2013). The sequential propensity household projection model. *Demographic Research,**28*, 681–712. 10.4054/DemRes.2013.28.24

[CR71] Yang, C., & Zeng, Y. (2000). Household projections for Taiwan. *Taiwanese Journal of Sociology,**24*, 239–279. in Chinese.

[CR72] Zeng, Y., Zhang, J., Tang, W., Mo, Y., Sun, Y., Li, M, Zhu, S., Grundy, E, Murphy, M., Bryant, J., Chao, F., Lutz, M., Lynch, S. M., Banks, D., Land, K.C., Feng, Q., & Wang, Z. (2024). Illustrative applications of the Bayesian model to estimate and probabilistically project total marriage rates and total divorce rates for probabilistic households and living arrangement projections. Manuscript submitted to peer-reviewed SCI journal for consideration of publication (available upon request).

[CR73] Zeng, Y. (1986). Changes in family structure in China: A simulation study. *Population and Development Review,**12*, 675–703. 10.2307/1973431

[CR74] Zeng, Y. (1991). *Family dynamics in China: A life table analysis*. The University of Wisconsin Press.

[CR75] Zeng, Y. (2007). Option for fertility policy transition in China. *Population and Development Review,**33*(2), 215–246. 10.1111/j.1728-4457.2007.00168.x

[CR76] Zeng, Y. (2011). Effects of demographic and retirement-age policies on future pension deficits, with an application to China. *Population and Development Review,**37*(3), 553–569. 10.1111/j.1728-4457.2011.00434.x 22167815 10.1111/j.1728-4457.2011.00434.x

[CR77] Zeng, Y., Chen, H., Wang, Z., & Land, K. C. (2013b). Implications of changes in households and living arrangements on future home-based care costs for disabled elders in China. *Journal of Aging and Health,**27*(3), 519–550. 10.1177/0898264314552690 10.1177/0898264314552690 PMC5099123 25213460

[CR78] Zeng, Y., Coale, A., Choe, M. K., Liang, Z., & Liu, L. (1994). Leaving parental home: Census based estimates for China, Japan, South Korea, the United States, France, and Sweden. *Population Studies,**48*(1), 65–80.

[CR79] Zeng, Y., Land, K. C., Gu, D., & Wang, Z. (2014). *Household and living arrangement projections: the extended cohort-component method and applications to the U.S. and China*. Springer Publisher.

[CR80] Zeng, Y., Land, K. C., Wang, Z., & Gu, D. (2006). U.S. family household momentum and dynamics: An extension and application of the ProFamy method. *Population Research and Policy Review,**25*(1), 1–41. 10.1007/s11113-006-7034-9

[CR81] Zeng, Y., Land, K. C., Wang, Z., & Gu, D. (2013a). Household and living arrangements projections at the sub-national level: An extended cohort-component approach. *Demography,**50*, 827–852. 10.1007/s13524-012-0171-3 23208782 10.1007/s13524-012-0171-3 PMC3622161

[CR82] Zeng, Y., Li, L., Wang, Z., Huang, H., & Norris, J. (2013c). Effects of changes in household structure on future housing demand in Hebei province, China. *Genus,**69*(2), 85–111.

[CR83] Zeng, Y., Morgan, P., Wang, Z., Gu, D., & Yang, C. (2012). A multistate life table analysis of union regimes in the united states– trends and racial differentials, 1970–2002. *Population Research and Policy Review,**31*, 207–234. 10.1007/s11113-011-9217-2 10.1007/s11113-011-9217-2 PMC3810991 24179311

[CR84] Zeng, Y., Vaupel, J. W., & Wang, Z. (1998). Household projection using conventional demographic data. *Population and Development Review,**S24*, 59–87. 10.2307/2808051

[CR85] Zeng, Y., Wang, Z., Jiang, L., & Gu, D. (2008). Future trend of family households and elderly living arrangement in China. *Genus,**64*(1/2), 9–36.

[CR86] Zeng, Y., Wang, Z., Ma, Z., & Chen, C. (2000). A simple method for estimating α and β: An extension of brass relational Gompertz fertility model. *Population Research and Policy Review,**19*(6), 525–549. 10.1023/A:1010695000412

[CR87] Zeng, Y., Yang, H., Wang, Z., & Li, L. (2021). Impacts of family household dynamics on residential energy demands in Hebei province of China. *Genus,**77*, 35. 10.1186/s41118-021-00148-0

[CR88] Zhang, J., Bryant, J., Chao, F., Banks, D., and Zeng, Y. (2024). Bayesian models for estimating and probabilistically projecting Total Marriage Rates (TMR(t)) and Total Divorce Rates (TDR(t)); manuscript under preparations

[CR89] Zhu, Y. (2021). Projections of size/structure of households ffinancial assets and liabilities in China. In Yi. Zeng (Ed.), *The innovative multistate methods for household and living arrangement projections and applications. Chapter 23. *Beijing: Science Press. in Chinese.

